# Novel Reassortant Clade 2.3.4.4 Avian Influenza A(H5N8) Virus in Wild Aquatic Birds, Russia, 2016

**DOI:** 10.3201/eid2302.161252

**Published:** 2017-02

**Authors:** Dong-Hun Lee, Kirill Sharshov, David E. Swayne, Olga Kurskaya, Ivan Sobolev, Marsel Kabilov, Alexander Alekseev, Victor Irza, Alexander Shestopalov

**Affiliations:** US Department of Agriculture, Athens, Georgia, USA (D.-H. Lee, D.E. Swayne);; Research Institute of Experimental and Clinical Medicine, Novosibirsk, Russia (K. Sharshov, O. Kurskaya, I. Sobolev, A. Alekseev, A. Shestopalov);; Institute of Chemical Biology and Fundamental Medicine SB RAS, Novosibirsk (M. Kabilov);; Federal Centre for Animal Health, Vladimir, Russia (V. Irza)

**Keywords:** Highly pathogenic avian influenza virus, HPAI, reassortment, Russia, Siberia, wild aquatic birds, phylogenetic analysis, clade 2.3.4.4, influenza in birds, bird diseases, viruses, influenza

## Abstract

The emergence of novel avian influenza viruses in migratory birds is of concern because of the potential for virus dissemination during fall migration. We report the identification of novel highly pathogenic avian influenza viruses of subtype H5N8, clade 2.3.4.4, and their reassortment with other avian influenza viruses in waterfowl and shorebirds of Siberia.

Highly pathogenic avian influenza virus (HPAIV) subtype H5N1 was first isolated from a goose in 1996 in Guangdong China (Gs/GD). This virus evolved into multiple hemagglutinin (HA) genetic clades and underwent reassortment with different neuraminidase and internal genes to generate subtype H5N8 clade 2.3.4.4 Gs/GD HPAIV, which first appeared in an outbreak in poultry in China in 2013 (*1*), followed closely by outbreaks in South Korea in January 2014 (*2*). During these outbreaks, 2 distinct groups of H5N8 viruses were identified; group A (Buan-like) and group B (Gochang-like). There have been no further reports of group B virus since its original detection in China and South Korea during 2014 (*3**,**4*). In contrast, in early 2014, group A viruses predominated in South Korea (*5*) and in September of that year were subsequently isolated from a Eurasian wigeon (*Anas penelope*) in Sakha Republic in northeast Siberia (*6*). On the basis of aquatic bird migration patterns, we hypothesized that HPAIV (H5N8) reached Siberia during the 2014 spring bird migration (*7*). The virus was probably carried by birds from Siberia to various countries of Asia, Europe, and North America during the fall migration, representing an intercontinental group A (icA) (*7*). We report detection of novel HPAIV (H5N8) from wild aquatic birds sampled in western Siberia during the summer of 2016.

In June 2016, we collected samples from 13 dead and 30 hunter-harvested wild aquatic birds around Uvs-Nuur Lake (Tyva Republic) at the Russia–Mongolia border. We isolated a total of 11 subtype H5 influenza viruses from birds of various species: the black-headed gull (*Larus ridibundus*), gray heron (*Ardea cinerea*), common tern (*Sterna hirundo*), great crested grebe (*Podiceps cristatus*), and great cormorant (*Phalacrocorax carbo*) (Technical Appendix Table 1). We characterized 3 of the viruses—A/great crested grebe/Uvs-Nuur Lake/341/2016(H5N8), A/common tern/Uvs-Nuur Lake/26/2016(H5N8), and A/gray heron/Uvs-Nuur Lake/20/2016(H5N8)—by sequencing, phylogenetic analysis, and intravenous pathogenicity index (IVPI) testing (online Technical Appendix).

We confirmed that all 3 isolates were HPAIV on the basis of amino acid sequence at the HA proteolytic cleavage site (PLREKRRKR/G) and individual IVPIs of 2.75-2.84 in chickens (Technical Appendix Table 1). The 3 isolates shared 99.2%–100% nucleotide identity across all 8 genes: HA, neuraminidase (NA), polymerase basic 2 (PB2), polymerase basic 2 (PB1), polymerase acidic (PA), nucleoprotein (NP), matrix (M), and nonstructural (NS). BLAST (https://www.ncbi.nlm.nih.gov/blast/) search results showed that the isolates shared >98% identity with low pathogenicity avian influenza virus (LPAIV) from Mongolia and China over 5 gene segments (PB1, PB2, PA, NP, and M) and >98.5% identity with the 2014 H5N8 clade 2.3.4.4 group B HPAIV for the remaining 3 gene segments (HA, NA, and NS) (Table). Phylogenetic analysis showed that the HA, NA, and NS genes clustered with H5N8 clade 2.3.4.4 group B HPAIV viruses identified in eastern China in 2014 (Technical Appendix Figure). The PB1, PB2, PA, NP, and M genes clustered with LPAIV identified in Mongolia, China, and Vietnam. 

**Table:** Nucleotide identity of near homologs in GenBank to the influenza A(H5N8) virus from Uvs-Nuur Lake, Russia, as of June 30, 2016*

Gene	Virus	Classification	% Identity
PB2	A/duck/Mongolia/30/2015(H3N8)	Eurasian LPAI	98.7
PB1	A/chicken/Hunan/S1267/2010(H4N6)	Eurasian LPAI	98.1
PA	A/duck/Mongolia/996/2015(H3N8)	Eurasian LPAI	98.7
HA	A/duck/eastern China/S1109/2014(H5N8)	H5N8 clade 2.3.4.4	99.1
NP	A/duck/Mongolia/129/2015(H3N3)	Eurasian LPAI	98.7
NA	A/duck/eastern China/S1109/2014(H5N8)	H5N8 clade 2.3.4.4	98.9
M	A/duck/Mongolia/179/2015(H3N8)	Eurasian LPAI	98.5
NS	A/duck/eastern China/S1109/2014(H5N8)	H5N8 clade 2.3.4.4	99.3

*HA, hemagglutinin; LPAI, low pathogenicity avian influenza; MP, matrix; NA, neuraminidase; NP, nucleoprotein; NS, nonstructural; PA, polymerase acidic; PB, polymerase basic.

Wild aquatic birds migrate to and congregate in Siberian wetlands for breeding and molting. Major wild aquatic bird migration routes overlap in Siberia, connecting this broad geographic area to the wintering grounds of Eurasia and Africa. This unique ecosystem has been implicated as a pathway for the dissemination of HPAIV during southward autumn migration of waterfowl, as seen in the spread of H5N1 clade 2.2 in 2005–2006 (*8*) and H5N8 clade 2.3.4.4 in 2014 (*6**,**7*). Uvs-Nuur Lake is a key habitat for 46 resident waterfowl species and 215 kinds of birds migrating south from Siberia (*9*). During widespread dissemination of the HPAIV clade 2.2 in 2006 and clade 2.3.2 in 2009, these viruses were also detected from wild aquatic birds at Uvs-Nuur Lake, suggesting this area is a useful site for surveillance of HPAIV in wild aquatic birds (*10*). Because numerous species of migratory shorebirds and waterfowl use the summer breeding grounds of Siberia, the identification of HPAIV infection in wild aquatic birds in this area signifies the potential for wide dissemination of these novel reassortant Group B H5N8 viruses during the 2016 fall migration.

Technical Appendix Supplemental materials and methods, influenza test results of wild birds from Uvs-Nurr Lake, Russia, phylogenetic tree of highly pathogenic and low pathogenicity influenza viruses, and acknowledgment of GISAID submitters.
