# Supplementary material for: Novel Reassortant Clade 2.3.4.4 Avian Influenza A(H5N8) Virus in Wild Aquatic Birds, Russia, 2016
Source: Emerg Infect Dis. 2017 Feb;23(2):359–60. doi: 10.3201/eid2302.161252 (PMC5324796; doi:10.3201/eid2302.161252)
Supplement: Technical Appendix — Supplemental materials and methods, influenza test results of wild birds from Uvs-Nurr Lake, Russia, phylogenetic tree of highly pathogenic and low pathogenicity influenza viruses, and acknowledgment of GISAID submitters. [file 16-1252-Techapp-s1.pdf]

Article DOI: http://dx.doi.org/10.3201/eid2302.161252

Novel Reassortant Clade 2.3.4.4 Avian Influenza A (H5N8) Virus in Wild Aquatic Birds, Russia, 2016

Technical Appendix

Materials and Methods

Samples

We collected 13 dead and 30 hunter harvested wild birds in the surroundings of Uvs-Nurr Lake (Tyva Republic) located at the Russia-Mongolia border in June 2016. As shown in appendix table 1, a total of 11 H5 viruses were isolated and included viruses obtained from black-headed gull (*Larus ridibundus*), grey heron (*Ardea cinerea*), common tern (*Sterna hirundo*), great crested grebe (*Podiceps cristatus*), and great cormorant (*Phalacrocorax carbo*) by chicken embryo inoculation using 10-day-old chicken embryonating eggs. All viruses caused the death of chicken embryos within 2 days. Isolates were confirmed to be H5 positive by AmpliSens Influenza virus A H5N1-FRT PCR kit (AmpliSens, Russia).

Genome sequencing and phylogenetic analysis

Complete genome sequencing of A/great crested grebe/Uvs-Nuur Lake/341/2016(H5N8), A/common tern/Uvs-Nuur Lake/26/2016(H5N8), and A/grey heron/Uvs-Nuur Lake/20/2016(H5N8) viruses was performed by next-generation sequencing using the Illumina MiSeq sequencer and Nextera XT DNA Library Preparation kit (Illumina) according to manufacturer`s instructions. The data were analyzed using CLC Genomics Workbench 8.5 (Qiagen, Redwood City, CA). Nucleotide sequences have been deposited in GISAID under no. EPI_ISL_224580, EPI_ISL_234057, and EPI_ISL_234058. We reconstructed the phylogenetic trees using selected representative sequences of Group icA and B and sequences sharing high nucleotide similarity (>98%) available in the GenBank and GISAID. Maximum-likelihood phylogenies for each of the gene segments were generated with RAxML (1) using the general time reversible (GTR) nucleotide substitution model, with among-site rate variation modeled

using a discrete gamma distribution. Bootstrap support values were generated using 1,000 rapid bootstrap replicates.

Intravenous pathogenicity index (IVPI)

For the intravenous pathogenicity index test of 3 viruses, 0.1 ml of 1:10 dilutions of infectious allantoic fluids were inoculated intravenously into ten 6-week-old specific pathogen free chickens. The IVPI was calculated according to the OIE standard protocol (available at: http://www.oie.int/international-standard-setting/terrestrial-code/) and isolates with an IVPI > 1.2 were determined to be HPAI. The challenge study and all experiments with live viruses were conducted in a biosafety level 3 facility.

References

1. Stamatakis A. RAxML version 8: a tool for phylogenetic analysis and post-analysis of large phylogenies. Bioinformatics. 2014;30:1312–3.

Technical Appendix Table 1. Summary of influenza test results of 11 wild birds from Uvs-Nurr Lake, Russia, 2016*

No.	Species	Sample	Status
1	Grey Heron (*Ardea cinerea*)	Dead (intestine)	rRT-PCR and isolation positive
2	Grey Heron (*Ardea cinerea)*	Dead (intestine)	rRT-PCR and isolation positive; IVPI=2.78; Complete genome sequencing
3	Common Tern (*Sterna hirundo)*	Dead (trachea)	rRT-PCR and isolation positive; IVPI=2.75; Complete genome sequencing
4	Common Tern (*Sterna hirundo)*	Dead (intestine)	rRT-PCR and isolation positive
5	Great Crested Grebe (*Podiceps cristatus)*	Dead (trachea)	rRT-PCR and isolation positive; IVPI=2.84; Complete genome sequencing
6	Black-headed Gull (*Larus ridibundus)*	Dead (trachea)	rRT-PCR and isolation positive
7	Black-headed Gull (*Larus ridibundus)*	Dead (trachea)	rRT-PCR and isolation positive
8	Great Cormorant (*Phalacrocorax carbo)*	Dead (brain)	rRT-PCR and isolation positive
9	Great Cormorant (*Phalacrocorax carbo)*	Hunter harvested (cloacal swab)	rRT-PCR and isolation positive
10	Great Cormorant (*Phalacrocorax carbo)*	Hunter harvested (cloacal swab)	rRT-PCR and isolation positive
11	Black-headed Gull (*Larus ridibundus)*	Hunter harvested (cloacal swab)	rRT-PCR and isolation positive

*rRT-PCR: real-time reverse transcription polymerase chain reaction

Technical Appendix Table 2. GISAID submitters for influenza virus segments used in this study*

Segment ID	Segment	Country	Collection date	Isolate name	Submitting Lab
EPI595116	HA	Korea, Republic of	2014-Dec-24	A/greater white-fronted goose/Korea/K14-367-4/2014	Konkuk University
EPI576391	HA	Sweden	2015-Mar-05	A/MuteSwan/Sweden/SVA-1503130141-SZ543/2015	National Veterinary Institute
EPI544756	HA	Germany	2014-Nov-04	A/turkey/Germany-MV/R2472/2014	Friedrich-Loeffler-Institut
EPI553208	HA	Japan	2014-Nov-23	A/crane/Kagoshima/KU1/2014	Kagoshima University
EPI573664	HA	Japan	2015-Jan-03	A/crane/Kagoshima/KU53/2015(H5N8)	Kagoshima University
EPI553362	HA	Japan	2014-Dec-01	A/environment/Kagoshima/KU-ngr-H/2014	Kagoshima University
EPI573638	HA	Japan	2014-Dec-07	A/crane/Kagoshima/KU13/2014(H5N8)	Kagoshima University
EPI553349	HA	Russian Federation	2014-Sep-25	A/wigeon/Sakha/1/2014	State Research Center of Virology and Biotechnology Vector
EPI547678	HA	Netherlands	2014-Nov-14	A/Chicken/Netherlands/14015526/2014	Central Veterinary Institute
EPI547673	HA	United Kingdom	2014-Nov-14	A/duck/England/36254/14	Animal and Plant Health Agency (APHA)
EPI550848	HA	United Kingdom	2014-Nov-14	A/duck/England/36038/14	Animal and Plant Health Agency (APHA)
EPI573672	HA	Japan	2015-Jan-14	A/mallard duck/Kagoshima/KU70/2015(H5N8)	Kagoshima University
EPI573680	HA	Japan	2015-Feb-13	A/mallard duck/Kagoshima/KU116/2015(H5N8)	Kagoshima University
EPI595094	HA	Korea, Republic of	2014-Dec-24	A/mandarin duck/Korea/K14-366-1/2014	Konkuk University

*We acknowledge the authors, originating and submitting laboratories of the sequences from GISAID's EpiFlu™ Database on which this research is based. Contact details of submitters can be found at: http://platform.gisaid.org/epi3/frontend#39414f

Technical Appendix Figure (following pages). Maximum likelihood phylogenetic trees for the (A) hemagglutinin (HA), (B) neuraminidase (NA), (C) polymerase basic-2 (PB2), (D) polymerase basic-1 (PB1), (E) polymerase acidic (PA), (F) nucleoprotein (NP), (G) matrix (MP), and (H) nonstructural (NS) gene segments for avian influenza virus isolates from Russia and reference isolates. Highly pathogenic and low pathogenic influenza virus sequences from Genbank (https://www.ncbi.nlm.nih.gov/genbank/) and the GISAID EpiFlu™ database (http://platform.gisaid.org/epi3/frontend#39414f) were used for each phylogenetic comparison. The genetic clusters of highly pathogenic avian influenza viruses are annotated by brackets to the right of the tree. At each branch, the number indicates a bootstrap value (>70%). Black circles indicate the H5N8 viruses sequenced in this study. Scale bar indicates nucleotide substitutions per site.

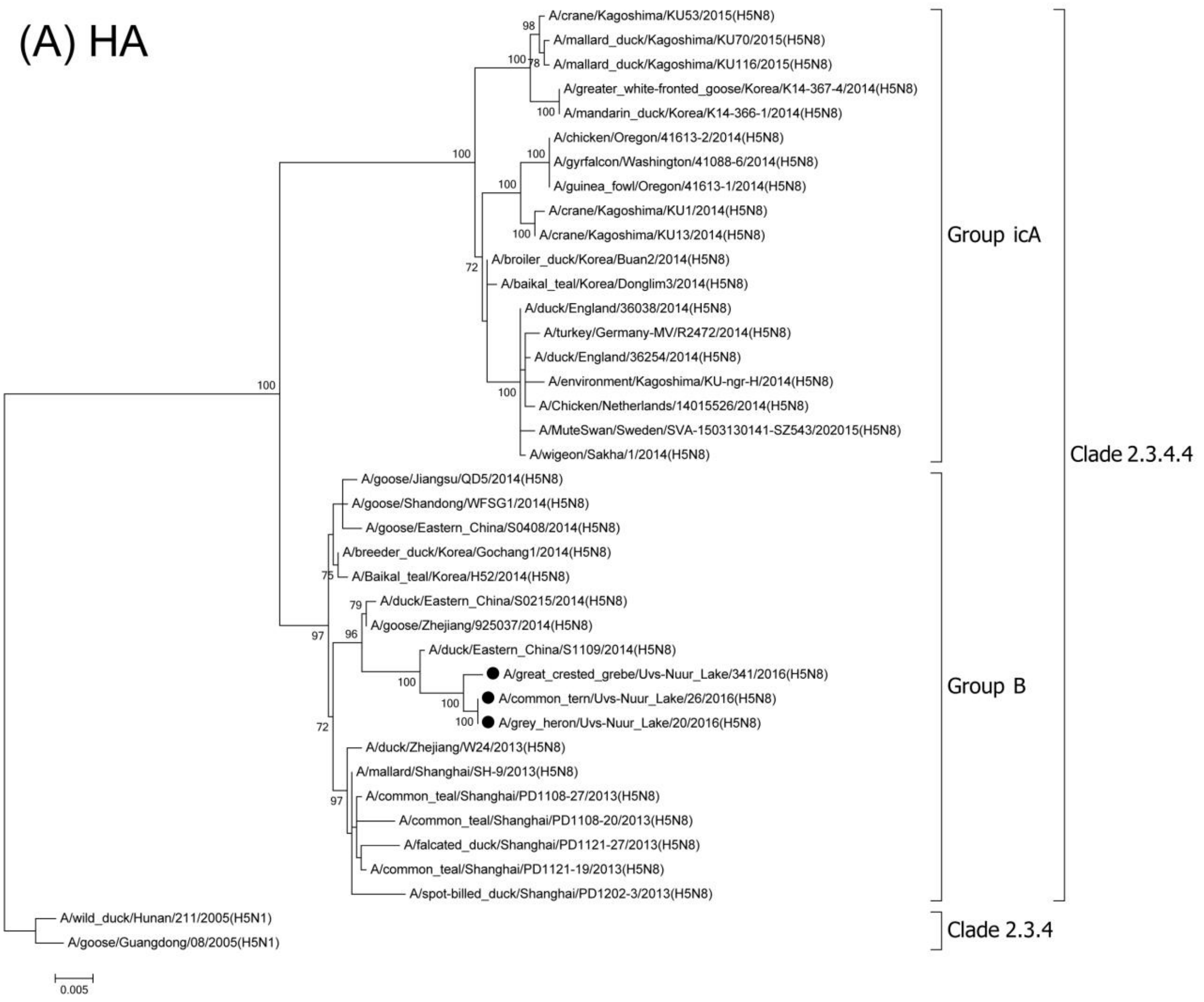

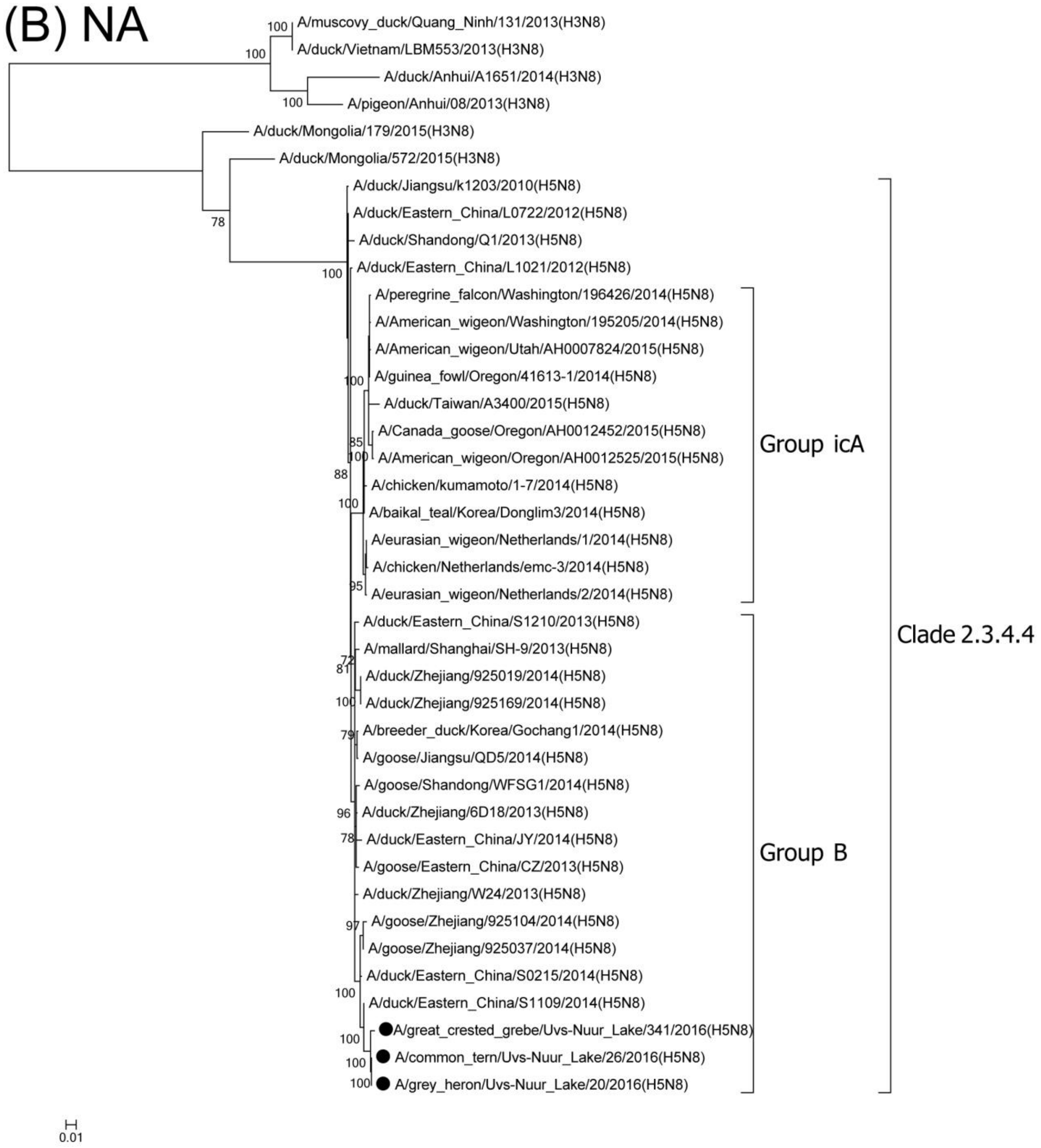
(B) NA
A/muscovy_duck/Quang_Ninh/131/2013(H3N8)
A/duck/Vietnam/LBM553/2013(H3N8)
A/duck/Anhui/A1651/2014(H3N8)
A/pigeon/Anhui/08/2013(H3N8)
A/duck/Mongolia/179/2015(H3N8)
A/duck/Mongolia/572/2015(H3N8)
A/duck/Jiangsu/k1203/2010(H5N8)
A/duck/Eastern_China/L0722/2012(H5N8)
A/duck/Shandong/Q1/2013(H5N8)
A/duck/Eastern_China/L1021/2012(H5N8)
A/peregrine_falcon/Washington/196426/2014(H5N8)
A/American_wigeon/Washington/195205/2014(H5N8)
A/American_wigeon/Utah/AH0007824/2015(H5N8)
A/guinea_fowl/Oregon/41613-1/2014(H5N8)
A/duck/Taiwan/A3400/2015(H5N8)
A/Canada_goose/Oregon/AH0012452/2015(H5N8)
A/American_wigeon/Oregon/AH0012525/2015(H5N8)
A/chicken/kumamoto/1-7/2014(H5N8)
A/baikal_teal/Korea/Donglim3/2014(H5N8)
A/eurasian_wigeon/Netherlands/1/2014(H5N8)
A/chicken/Netherlands/emc-3/2014(H5N8)
A/eurasian_wigeon/Netherlands/2/2014(H5N8)
A/duck/Eastern_China/S1210/2013(H5N8)
A/mallard/Shanghai/SH-9/2013(H5N8)
A/duck/Zhejiang/925019/2014(H5N8)
A/duck/Zhejiang/925169/2014(H5N8)
A/breeder_duck/Korea/Gochang1/2014(H5N8)
A/goose/Jiangsu/QD5/2014(H5N8)
A/goose/Shandong/WFSG1/2014(H5N8)
A/duck/Zhejiang/6D18/2013(H5N8)
A/duck/Eastern_China/JY/2014(H5N8)
A/goose/Eastern_China/CZ/2013(H5N8)
A/duck/Zhejiang/W24/2013(H5N8)
A/goose/Zhejiang/925104/2014(H5N8)
A/goose/Zhejiang/925037/2014(H5N8)
A/duck/Eastern_China/S0215/2014(H5N8)
A/duck/Eastern_China/S1109/2014(H5N8)
A/great_crested_grebe/Uvs-Nuur_Lake/341/2016(H5N8)
A/common_tern/Uvs-Nuur_Lake/26/2016(H5N8)
A/grey_heron/Uvs-Nuur_Lake/20/2016(H5N8)
Group icA
Group B
Clade 2.3.4.4
0.01

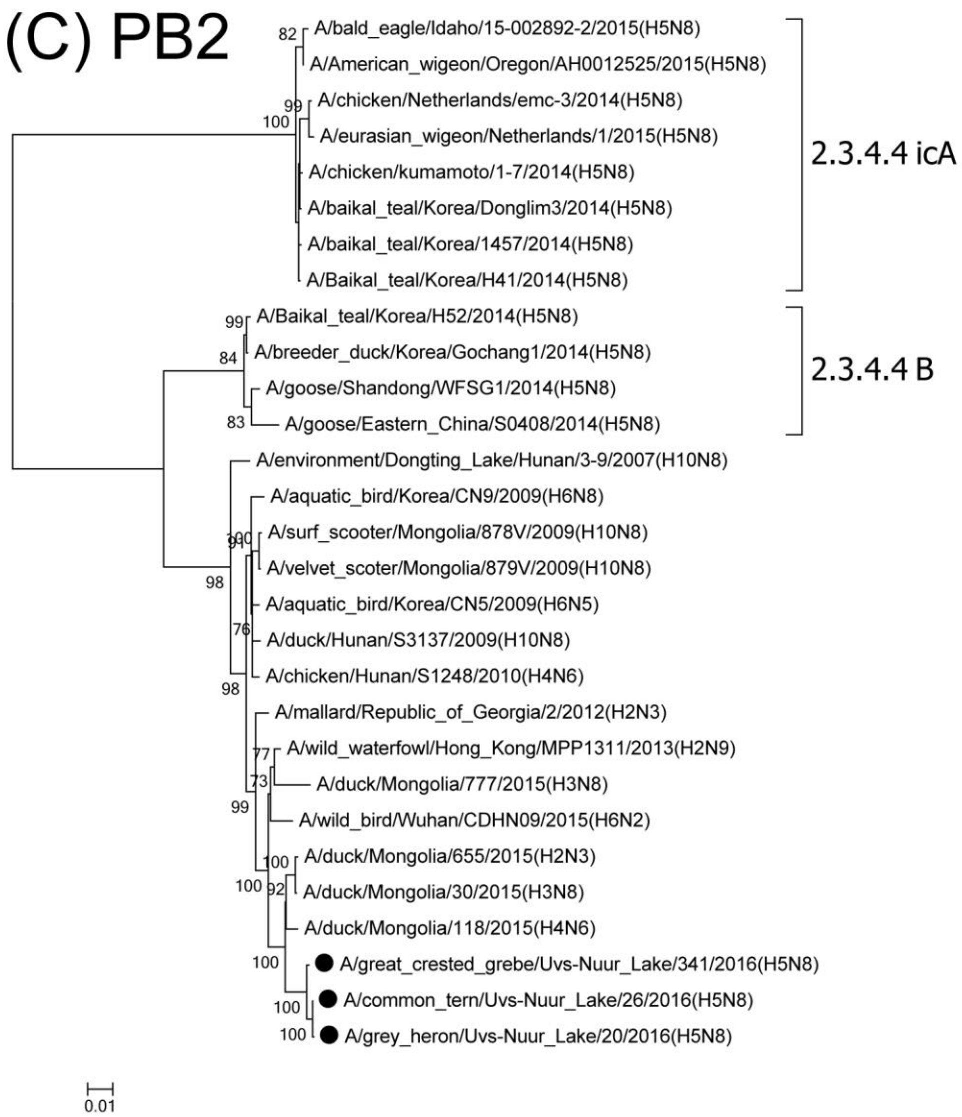
(C) PB2
A/bald_eagle/Idaho/15-002892-2/2015(H5N8)
A/American_wigeon/Oregon/AH0012525/2015(H5N8)
A/chicken/Netherlands/emc-3/2014(H5N8)
A/eurasian_wigeon/Netherlands/1/2015(H5N8)
A/chicken/kumamoto/1-7/2014(H5N8)
A/baikal_teal/Korea/Donglim3/2014(H5N8)
A/baikal_teal/Korea/1457/2014(H5N8)
A/Baikal_teal/Korea/H41/2014(H5N8)
2.3.4.4 icA
A/Baikal_teal/Korea/H52/2014(H5N8)
A/breeder_duck/Korea/Gochang1/2014(H5N8)
A/goose/Shandong/WFSG1/2014(H5N8)
A/goose/Eastern_China/S0408/2014(H5N8)
2.3.4.4 B
A/environment/Dongting_Lake/Hunan/3-9/2007(H10N8)
A/aquatic_bird/Korea/CN9/2009(H6N8)
A/surf_scooter/Mongolia/878V/2009(H10N8)
A/velvet_scoter/Mongolia/879V/2009(H10N8)
A/aquatic_bird/Korea/CN5/2009(H6N5)
A/duck/Hunan/S3137/2009(H10N8)
A/chicken/Hunan/S1248/2010(H4N6)
A/mallard/Republic_of_Georgia/2/2012(H2N3)
A/wild_waterfowl/Hong_Kong/MPP1311/2013(H2N9)
A/duck/Mongolia/777/2015(H3N8)
A/wild_bird/Wuhan/CDHN09/2015(H6N2)
A/duck/Mongolia/655/2015(H2N3)
A/duck/Mongolia/30/2015(H3N8)
A/duck/Mongolia/118/2015(H4N6)
A/great_crested_grebe/Uvs-Nuur_Lake/341/2016(H5N8)
A/common_tern/Uvs-Nuur_Lake/26/2016(H5N8)
A/grey_heron/Uvs-Nuur_Lake/20/2016(H5N8)
0.01

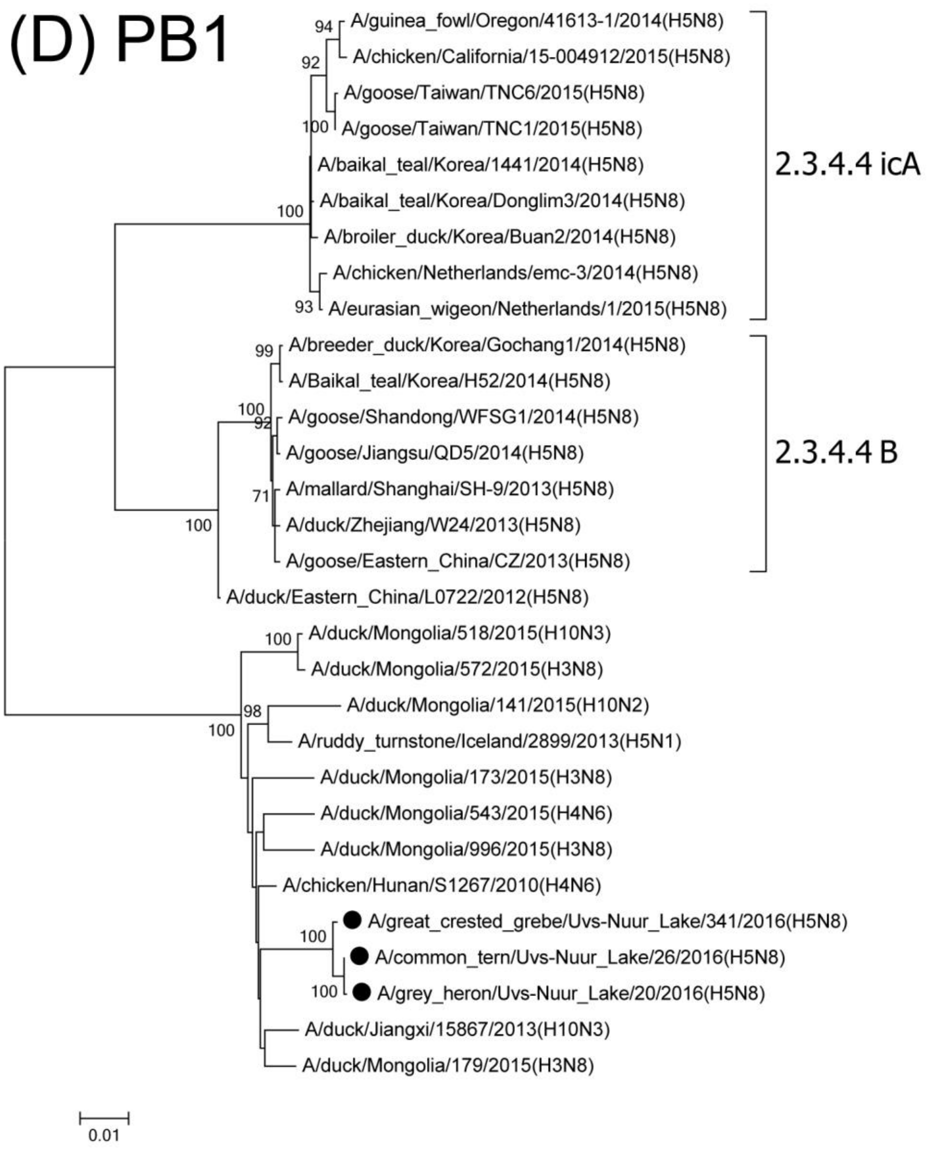

(D) PB1
A/guinea_fowl/Oregon/41613-1/2014(H5N8)
A/chicken/California/15-004912/2015(H5N8)
A/goose/Taiwan/TNC6/2015(H5N8)
A/goose/Taiwan/TNC1/2015(H5N8)
A/baikal_teal/Korea/1441/2014(H5N8)
A/baikal_teal/Korea/Donglim3/2014(H5N8)
A/broiler_duck/Korea/Buan2/2014(H5N8)
A/chicken/Netherlands/emc-3/2014(H5N8)
A/eurasian_wigeon/Netherlands/1/2015(H5N8)
2.3.4.4 icA
A/breeder_duck/Korea/Gochang1/2014(H5N8)
A/Baikal_teal/Korea/H52/2014(H5N8)
A/goose/Shandong/WFSG1/2014(H5N8)
A/goose/Jiangsu/QD5/2014(H5N8)
A/mallard/Shanghai/SH-9/2013(H5N8)
A/duck/Zhejiang/W24/2013(H5N8)
A/goose/Eastern_China/CZ/2013(H5N8)
2.3.4.4 B
A/duck/Eastern_China/L0722/2012(H5N8)
A/duck/Mongolia/518/2015(H10N3)
A/duck/Mongolia/572/2015(H3N8)
A/duck/Mongolia/141/2015(H10N2)
A/ruddy_turnstone/Iceland/2899/2013(H5N1)
A/duck/Mongolia/173/2015(H3N8)
A/duck/Mongolia/543/2015(H4N6)
A/duck/Mongolia/996/2015(H3N8)
A/chicken/Hunan/S1267/2010(H4N6)
A/great_crested_grebe/Uvs-Nuur_Lake/341/2016(H5N8)
A/common_tern/Uvs-Nuur_Lake/26/2016(H5N8)
A/grey_heron/Uvs-Nuur_Lake/20/2016(H5N8)
A/duck/Jiangxi/15867/2013(H10N3)
A/duck/Mongolia/179/2015(H3N8)
94
92
100
100
93
99
100
92
71
100
100
98
100
100
100
0.01

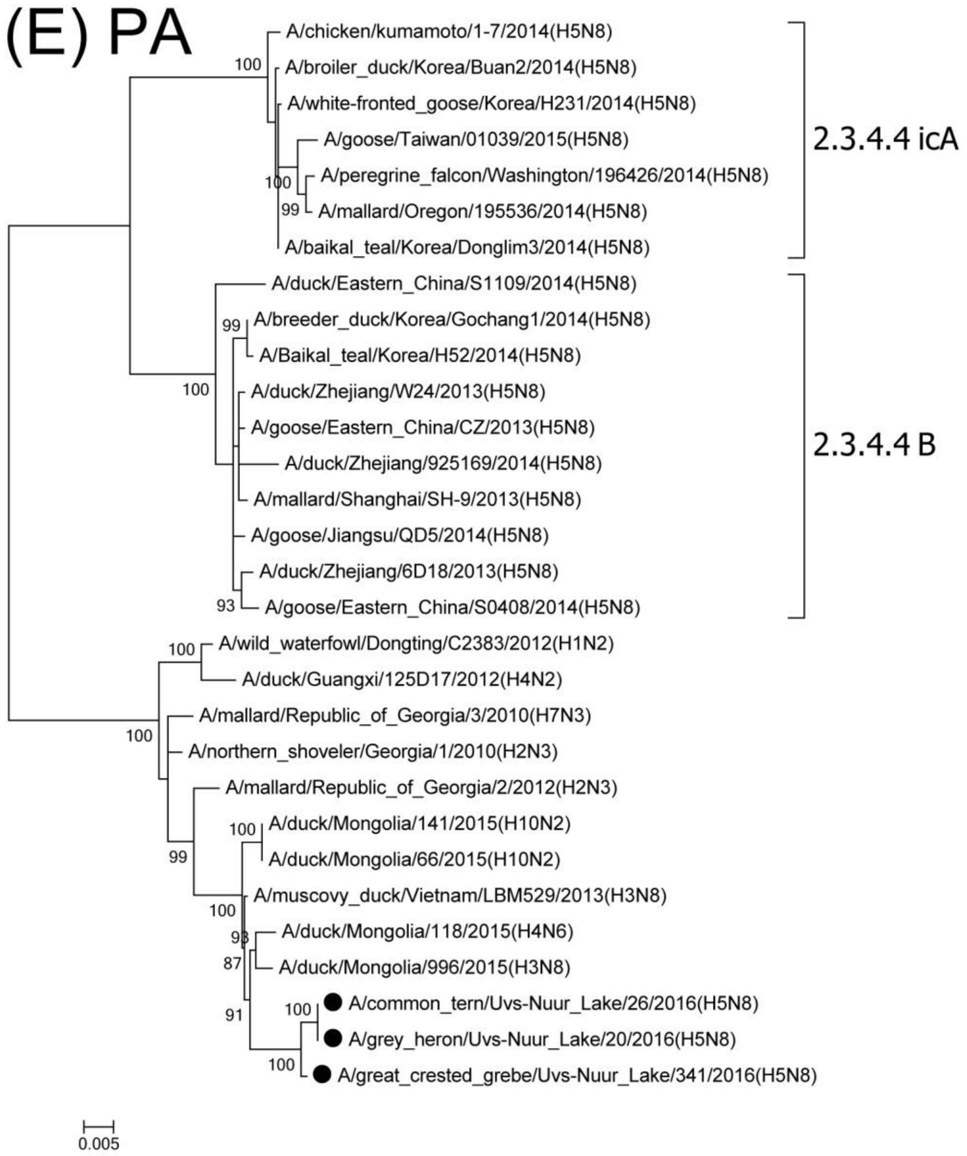

(E) PA
A/chicken/kumamoto/1-7/2014(H5N8)
A/broiler_duck/Korea/Buan2/2014(H5N8)
A/white-fronted_goose/Korea/H231/2014(H5N8)
A/goose/Taiwan/01039/2015(H5N8)
A/peregrine_falcon/Washington/196426/2014(H5N8)
A/mallard/Oregon/195536/2014(H5N8)
A/baikal_teal/Korea/Donglim3/2014(H5N8)
2.3.4.4 icA
A/duck/Eastern_China/S1109/2014(H5N8)
A/breeder_duck/Korea/Gochang1/2014(H5N8)
A/Baikal_teal/Korea/H52/2014(H5N8)
A/duck/Zhejiang/W24/2013(H5N8)
A/goose/Eastern_China/CZ/2013(H5N8)
A/duck/Zhejiang/925169/2014(H5N8)
A/mallard/Shanghai/SH-9/2013(H5N8)
A/goose/Jiangsu/QD5/2014(H5N8)
A/duck/Zhejiang/6D18/2013(H5N8)
A/goose/Eastern_China/S0408/2014(H5N8)
2.3.4.4 B
A/wild_waterfowl/Dongting/C2383/2012(H1N2)
A/duck/Guangxi/125D17/2012(H4N2)
A/mallard/Republic_of_Georgia/3/2010(H7N3)
A/northern_shoveler/Georgia/1/2010(H2N3)
A/mallard/Republic_of_Georgia/2/2012(H2N3)
A/duck/Mongolia/141/2015(H10N2)
A/duck/Mongolia/66/2015(H10N2)
A/muscovy_duck/Vietnam/LBM529/2013(H3N8)
A/duck/Mongolia/118/2015(H4N6)
A/duck/Mongolia/996/2015(H3N8)
A/common_tern/Uvs-Nuur_Lake/26/2016(H5N8)
A/grey_heron/Uvs-Nuur_Lake/20/2016(H5N8)
A/great_crested_grebe/Uvs-Nuur_Lake/341/2016(H5N8)
100
100
99
99
100
93
100
100
99
100
100
93
87
91
100
100
0.005

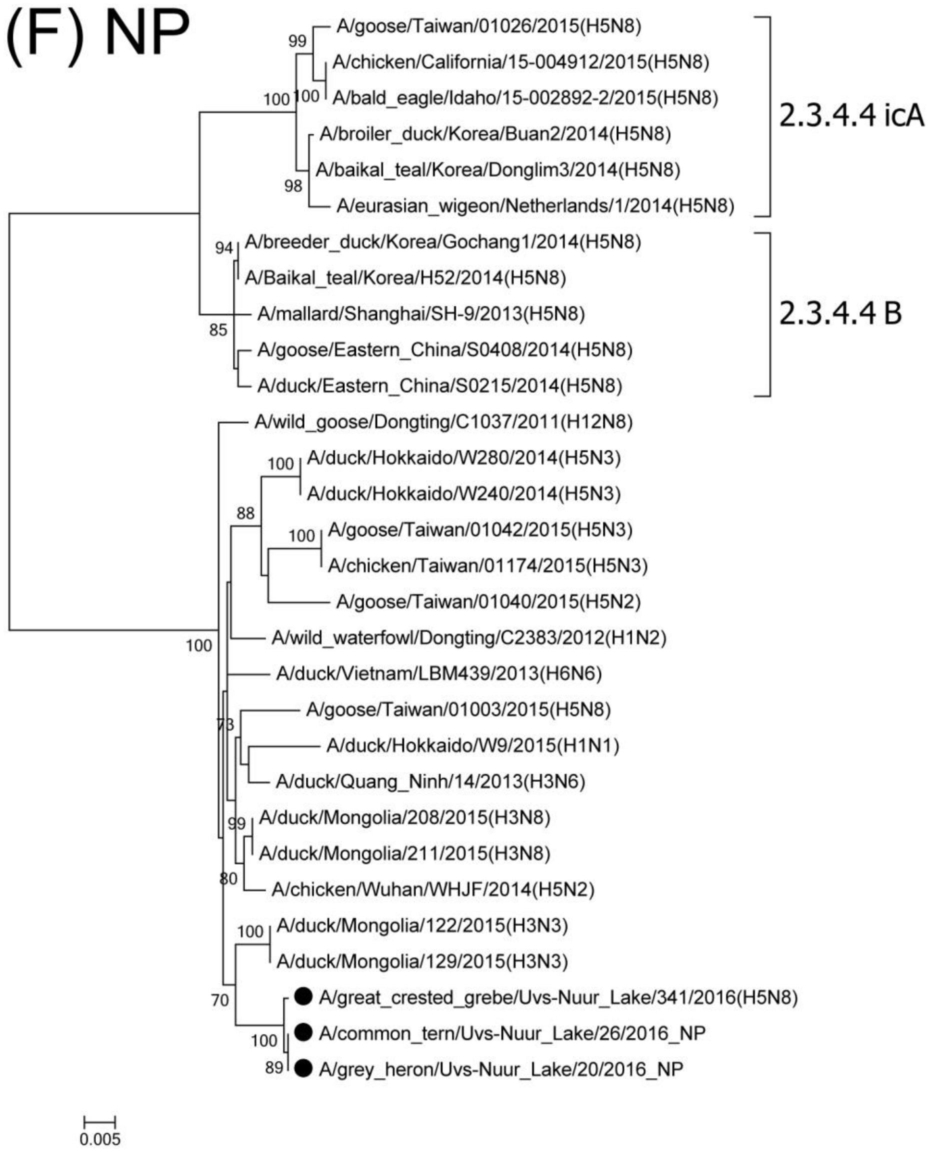
(F) NP
A/goose/Taiwan/01026/2015(H5N8)
A/chicken/California/15-004912/2015(H5N8)
A/bald_eagle/Idaho/15-002892-2/2015(H5N8)
A/broiler_duck/Korea/Buan2/2014(H5N8)
A/baikal_teal/Korea/Donglim3/2014(H5N8)
A/eurasian_wigeon/Netherlands/1/2014(H5N8)
2.3.4.4 icA
A/breeder_duck/Korea/Gochang1/2014(H5N8)
A/Baikal_teal/Korea/H52/2014(H5N8)
A/mallard/Shanghai/SH-9/2013(H5N8)
A/goose/Eastern_China/S0408/2014(H5N8)
A/duck/Eastern_China/S0215/2014(H5N8)
2.3.4.4 B
A/wild_goose/Dongting/C1037/2011(H12N8)
A/duck/Hokkaido/W280/2014(H5N3)
A/duck/Hokkaido/W240/2014(H5N3)
A/goose/Taiwan/01042/2015(H5N3)
A/chicken/Taiwan/01174/2015(H5N3)
A/goose/Taiwan/01040/2015(H5N2)
A/wild_waterfowl/Dongting/C2383/2012(H1N2)
A/duck/Vietnam/LBM439/2013(H6N6)
A/goose/Taiwan/01003/2015(H5N8)
A/duck/Hokkaido/W9/2015(H1N1)
A/duck/Quang_Ninh/14/2013(H3N6)
A/duck/Mongolia/208/2015(H3N8)
A/duck/Mongolia/211/2015(H3N8)
A/chicken/Wuhan/WHJF/2014(H5N2)
A/duck/Mongolia/122/2015(H3N3)
A/duck/Mongolia/129/2015(H3N3)
A/great_crested_grebe/Uvs-Nuur_Lake/341/2016(H5N8)
A/common_tern/Uvs-Nuur_Lake/26/2016_NP
A/grey_heron/Uvs-Nuur_Lake/20/2016_NP
0.005

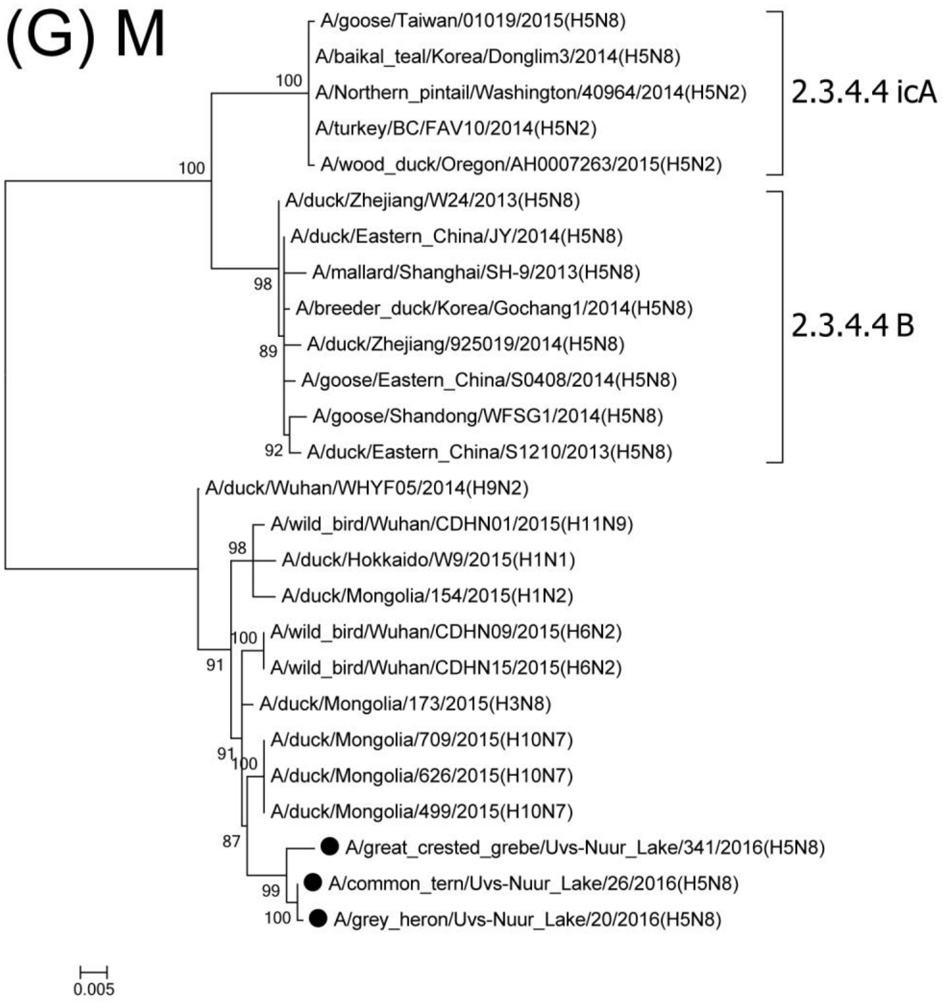
(G) M
A/goose/Taiwan/01019/2015(H5N8)
A/baikal_teal/Korea/Donglim3/2014(H5N8)
A/Northern_pintail/Washington/40964/2014(H5N2)
A/turkey/BC/FAV10/2014(H5N2)
A/wood_duck/Oregon/AH0007263/2015(H5N2)
2.3.4.4 icA
A/duck/Zhejiang/W24/2013(H5N8)
A/duck/Eastern_China/JY/2014(H5N8)
A/mallard/Shanghai/SH-9/2013(H5N8)
A/breeder_duck/Korea/Gochang1/2014(H5N8)
A/duck/Zhejiang/925019/2014(H5N8)
A/goose/Eastern_China/S0408/2014(H5N8)
A/goose/Shandong/WFSG1/2014(H5N8)
A/duck/Eastern_China/S1210/2013(H5N8)
2.3.4.4 B
A/duck/Wuhan/WHYF05/2014(H9N2)
A/wild_bird/Wuhan/CDHN01/2015(H11N9)
A/duck/Hokkaido/W9/2015(H1N1)
A/duck/Mongolia/154/2015(H1N2)
A/wild_bird/Wuhan/CDHN09/2015(H6N2)
A/wild_bird/Wuhan/CDHN15/2015(H6N2)
A/duck/Mongolia/173/2015(H3N8)
A/duck/Mongolia/709/2015(H10N7)
A/duck/Mongolia/626/2015(H10N7)
A/duck/Mongolia/499/2015(H10N7)
A/great_crested_grebe/Uvs-Nuur_Lake/341/2016(H5N8)
A/common_tern/Uvs-Nuur_Lake/26/2016(H5N8)
A/grey_heron/Uvs-Nuur_Lake/20/2016(H5N8)
0.005

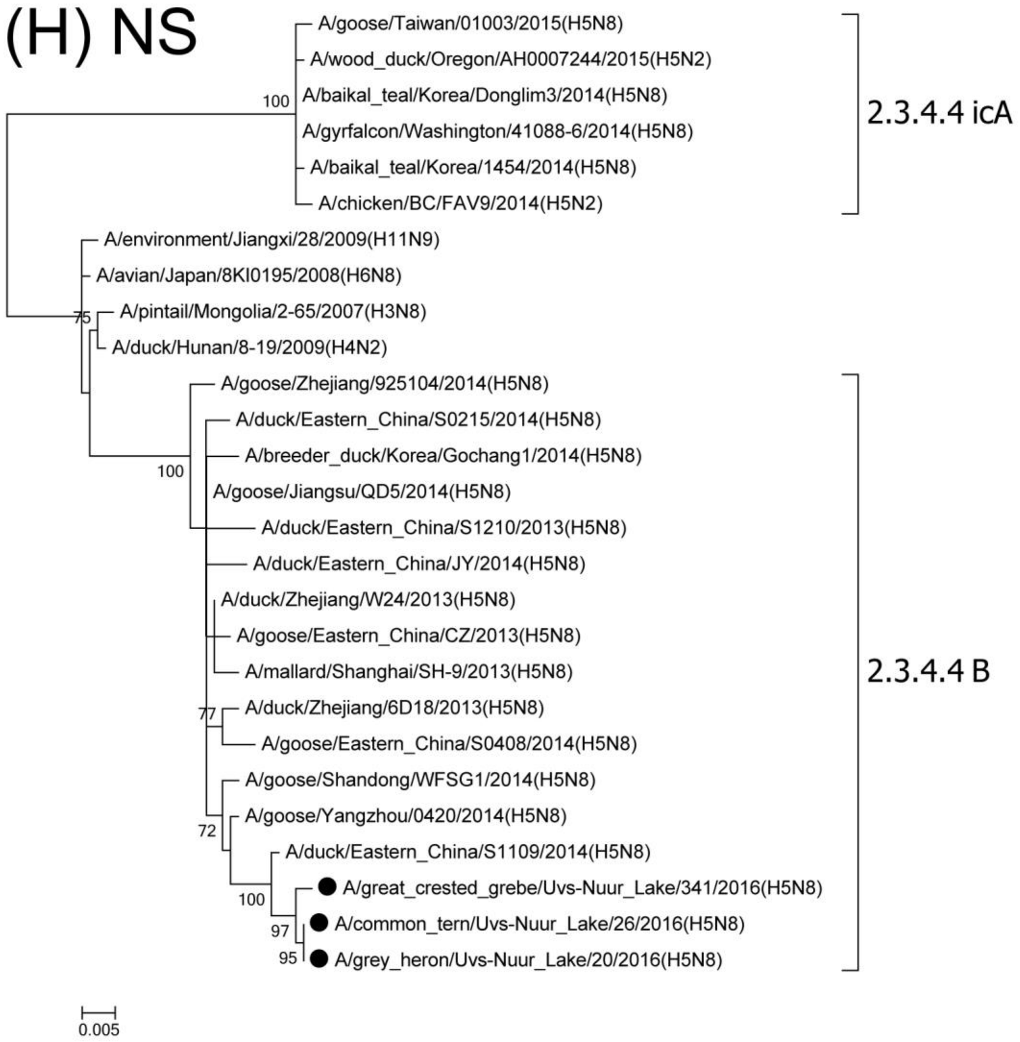
(H) NS
A/goose/Taiwan/01003/2015(H5N8)
A/wood_duck/Oregon/AH0007244/2015(H5N2)
100
A/baikal_teal/Korea/Donglim3/2014(H5N8)
A/gyrfalcon/Washington/41088-6/2014(H5N8)
A/baikal_teal/Korea/1454/2014(H5N8)
A/chicken/BC/FAV9/2014(H5N2)
2.3.4.4 icA
A/environment/Jiangxi/28/2009(H11N9)
A/avian/Japan/8KI0195/2008(H6N8)
75
A/pintail/Mongolia/2-65/2007(H3N8)
A/duck/Hunan/8-19/2009(H4N2)
A/goose/Zhejiang/925104/2014(H5N8)
A/duck/Eastern_China/S0215/2014(H5N8)
100
A/breeder_duck/Korea/Gochang1/2014(H5N8)
A/goose/Jiangsu/QD5/2014(H5N8)
A/duck/Eastern_China/S1210/2013(H5N8)
A/duck/Eastern_China/JY/2014(H5N8)
A/duck/Zhejiang/W24/2013(H5N8)
A/goose/Eastern_China/CZ/2013(H5N8)
A/mallard/Shanghai/SH-9/2013(H5N8)
77
A/duck/Zhejiang/6D18/2013(H5N8)
A/goose/Eastern_China/S0408/2014(H5N8)
A/goose/Shandong/WFSG1/2014(H5N8)
72
A/goose/Yangzhou/0420/2014(H5N8)
A/duck/Eastern_China/S1109/2014(H5N8)
100
A/great_crested_grebe/Uvs-Nuur_Lake/341/2016(H5N8)
97
A/common_tern/Uvs-Nuur_Lake/26/2016(H5N8)
95
A/grey_heron/Uvs-Nuur_Lake/20/2016(H5N8)
2.3.4.4 B
0.005
